# Reconsidering anion inhibitors in the general context of drug design studies of modulators of activity of the classical enzyme carbonic anhydrase

**DOI:** 10.1080/14756366.2021.1882453

**Published:** 2021-02-21

**Authors:** Alessio Nocentini, Andrea Angeli, Fabrizio Carta, Jean-Yves Winum, Raivis Zalubovskis, Simone Carradori, Clemente Capasso, William A. Donald, Claudiu T. Supuran

**Affiliations:** a Neurofarba Department, Pharmaceutical and Nutraceutical Section, University of Florence, Florence, Italy; b IBMM, Univ. Montpellier, CNRS, ENSCM, Montpellier, France; c Latvian Institute of Organic Synthesis, Riga, Latvia; d Institute of Technology of Organic Chemistry, Faculty of Materials Science and Applied Chemistry, Riga Technical University, Riga, Latvia; e Department of Pharmacy, "G. d'Annunzio" University of Chieti-Pescara, Chieti, Italy; f Institute of Biosciences and Bioresources, National Research Council, Napoli, Italy; g School of Chemistry, University of New South Wales, Sydney, Australia

**Keywords:** carbonic anhydrase, inhibitor, sulphonamide, inhibition mechanisms, cyanate

## Abstract

Inorganic anions inhibit the metalloenzyme carbonic anhydrase (CA, EC 4.2.1.1) generally by coordinating to the active site metal ion. Cyanate was reported as a non-coordinating CA inhibitor but those erroneous results were subsequently corrected by another group. We review the anion CA inhibitors (CAIs) in the more general context of drug design studies and the discovery of a large number of inhibitor classes and inhibition mechanisms, including zinc binders (sulphonamides and isosteres, dithiocabamates and isosteres, thiols, selenols, benzoxaboroles, ninhydrins, etc.); inhibitors anchoring to the zinc-coordinated water molecule (phenols, polyamines, sulfocoumarins, thioxocoumarins, catechols); CAIs occluding the entrance to the active site (coumarins and derivatives, lacosamide), as well as compounds that bind outside the active site. All these new chemotypes integrated with a general procedure for obtaining isoform-selective compounds (the tail approach) has resulted, through the guidance of rigorous X-ray crystallography experiments, in the development of highly selective CAIs for all human CA isoforms with many pharmacological applications.

## 1. Introduction

Carbonic anhydrase (CA, EC 4.2.1.1) was discovered almost a century ago by Meldrum and Roughton^1^ who described its efficient catalysis for the reversible hydration of blood CO_2_ to bicarbonate and protons. Subsequently, such a process was shown to be relevant physiologically for many systems in organisms throughout the phylogenetic tree^2^^,^^3^. Relevant discoveries on the catalytic and inhibition mechanisms of this enzyme were thereafter mainly done by a Swedish scientist, in the period from the 60 s to the 80 s, S. Lindskog, who proposed the catalytic mechanism that is still valid today^4^, i.e. the enzyme contains a nucleophilic zinc hydroxide species that can hydrate CO_2_ in a two-step, “ping-pong” process, as follows: (i) the attack of CO_2_ by the zinc hydroxide bound within the active site of the enzyme resulting in formation of bicarbonate, which is released into solution, and (ii) regeneration of the zinc hydroxide nucleophilic species by a proton transfer reaction from a zinc-coordinated water molecule to the reaction medium, which is the rate-determining step of the catalytic cycle^4^. The first investigation of anion CA inhibitors (CAIs) were also performed by Lindskog on both native and cobalt(II)-substituted enzymes^5^, demonstrating by means of electronic spectroscopic techniques (using the cobalt-enzyme) that such inhibitors coordinate to the catalytic metal ion in a tetrahedral or trigonal-bipyramidal geometry^3–5^. The first crystallographic studies of a CA isoform were also reported by researchers from Sweden (Lindskog’s and Nyman’s groups)^6^, whereas the first complete structure, that of human (h) isoform hCA II was published in 1972^7^.

Sulphonamides were reported to be CAIs in 1940 by Mann and Keilin^8^ who also reported some side effects that were observed with the newly discovered (in 1940) antibacterial drug sulphanilamide (4-amino-benzenesulfonamide). Krebs on the other hand published the first comprehensive structure-activity relationship (SAR) study of sulphonamide inhibitors^9^ and together with Roughton, one of the co-discoverers of the enzyme, proposed CA as “a tool in studying the mechanism of reactions involving H_2_CO_3_, CO_2_ or HCO_3_^−^”^10^, which is role that these enzymes still have today after thousands of valuable studies that have been performed worldwide^11–15^. This is probably due primarily to the fact that CAs are drug targets for a multitude of disorders, which are aspects that will be discussed later in this review.

Nowadays, eight CA genetic families are known in organisms from across the phylogenetic tree, more precisely α-, β-, γ-, δ-, ζ-, η-, θ- and ι-CAs^16–23^. Many representatives from these classes have been fully characterised and X-ray crystallographic structures are now known for several (and in some cases many) members of the α-, β-, γ-, ζ- and θ- classes^16–23^, which constitutes an excellent example of convergent evolution at the molecular level. However, the structures of δ-, η- and ι-CAs have not yet been reported to date.

**Figure 1.**
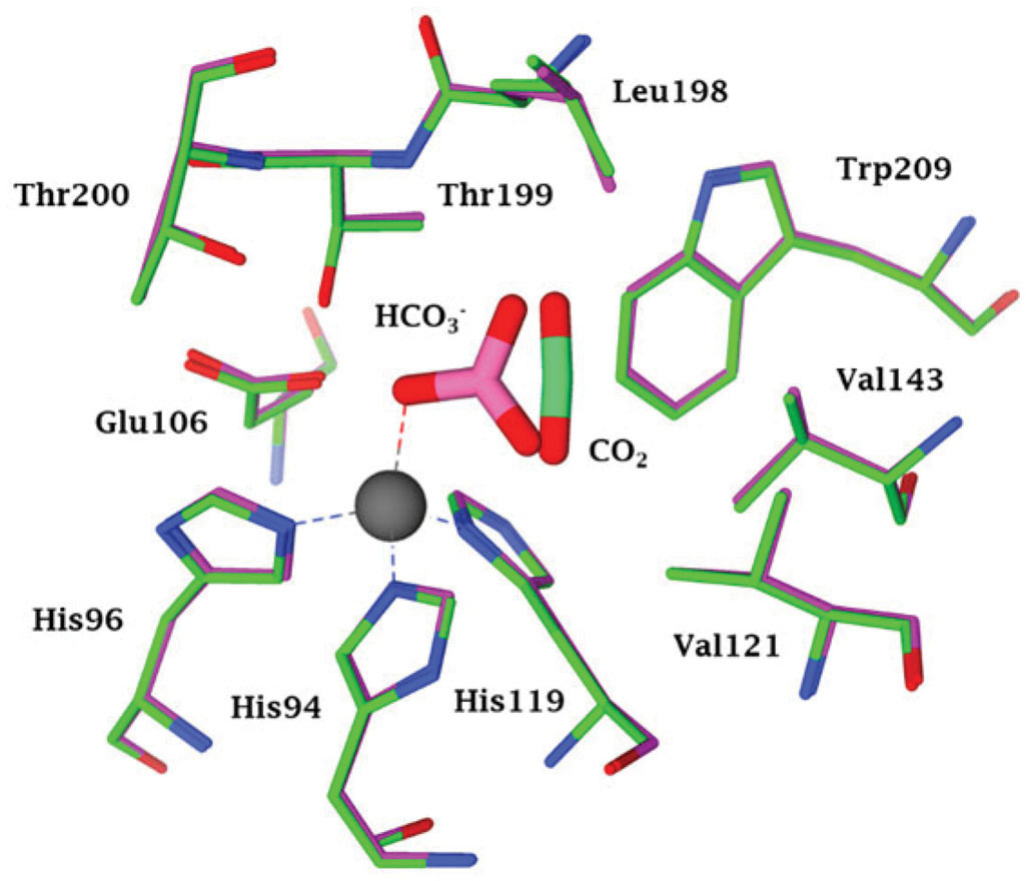
Active site view of the superimposed hCA II adducts with its main substrates: CO_2_ (green, pdb 2VVA) and bicarbonate (pink, pdb 2VVB) ^24^. The zinc ion is shown as a grey sphere with its three coordinated protein ligands (green/red), His94, 96 and 119. Amino acid residues involved in the binding of the substrates/inhibitors, at the bottom of the active site, are also shown.

Importantly, the progress in X-ray crystallography over the last few decades has revealed the elusive binding modes of CO_2_ and bicarbonate, which are the two main substrates of the enzyme^24^^,^^25^ (Figure 1). Indeed, as seen in Figure 1 for the α-class hCA II, CO_2_ is bound in a hydrophobic pocket that is lined with residues Val121, Vals143, Trp209 and Leu198 in close proximity (but not directly coordinated) to the zinc ion, whereas bicarbonate is coordinated to the metal ion in a monodentate fashion. Highly relevant studies from the groups of McKenna and Djinovic-Carugo^24^ based on high pressure and cryo-crystallographic data demonstrated that the substrate binding modes are probably similar in all CA classes. Such a conclusion was further supported by crystallographic structures of β-CAs by McKenna’s group^25^, in which the binding mode of CO_2_ to a β-CA expressed in the bacterium *Pseudomonas aeruginosa* (psCA3) was reported.

**Figure 2.**
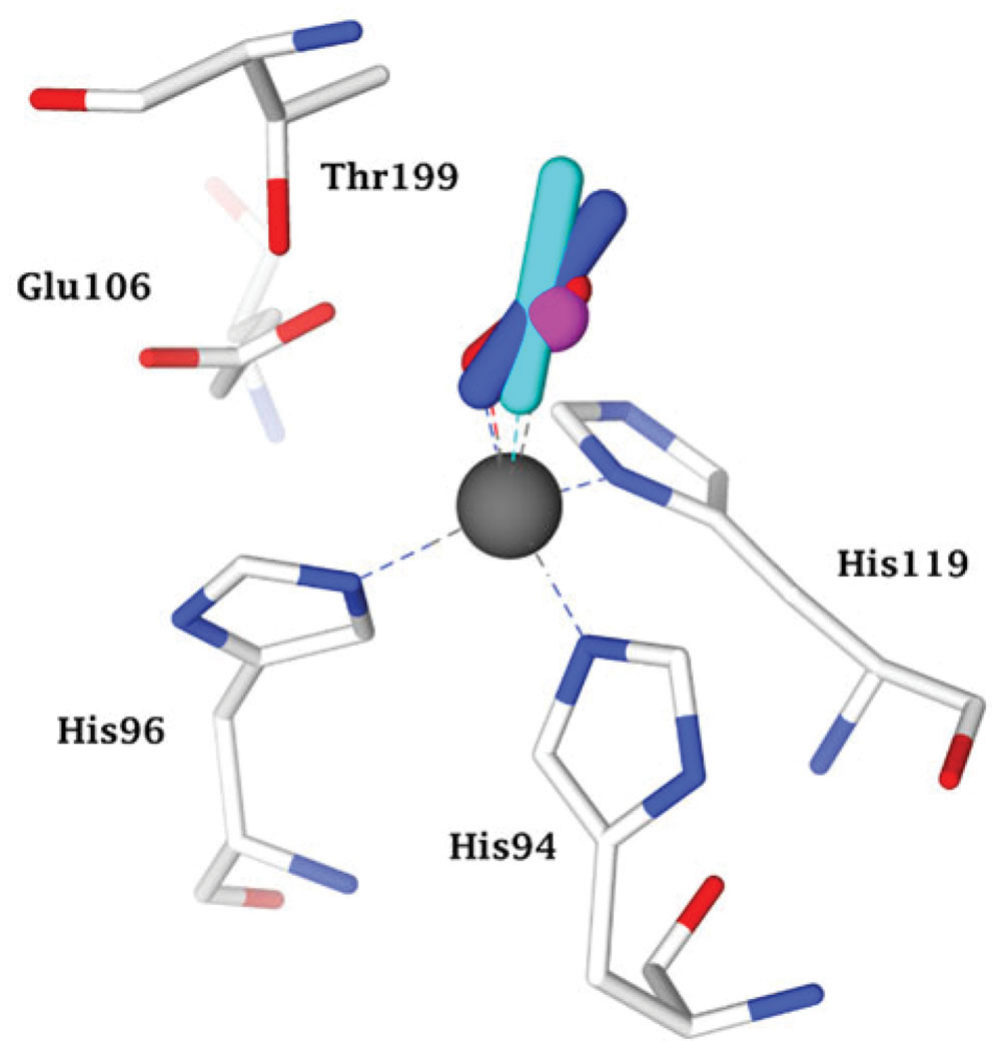
Representation of the superimposed X-ray crystallographic data for hCA II adducts with cyanate (cyan, pdb 4E5Q^31^) bromide (magenta, pdb 1RAZ^32^) azide (blue, pdb 1RAY^32^) O_2_ (presumably as superoxide anion or radical anion; red, pdb 5EOI^33^) with a tetrahedrally coordinated active site zinc ion (grey sphere). The key active site zinc ligands (His94, 96 and 119) and “gate-keeping” residues (Thr199, Glu106) are also shown.

## 2. Anion inhibitors

As mentioned above, Lindskog^3–5^ reported some of the first anion CA inhibition studies, considering the well-known fact that many (in)organic anions have a high affinity for complexing transition metal ions in solution or within the active sites of metalloenzymes^26–30^. Such pioneering studies in this field, before the X-ray crystallographic techniques became widely available (i.e. around 1995), used various biophysical techniques, such as circular dichroism, electron paramagnetic spectroscopy (EPR), electronic spectroscopy (UV/Vis), and NMR techniques together with metal-substituted CAs (incorporating Co(II), Cu(II), Mn(II), Ni(II), Cd(II) and other transition metal ions which have the desired spectral/paramagnetic properties, of which Zn^2+^, a d^10^ metal ion, is devoid). This allowed the investigation of the interactions of CA with various anions such as cyanide, cyanate, thiocyanate, azide, halides, carboxylates, hydrogen sulphide, and more complex anions (ferrocyanide, ferricyanide, etc.)^27–30^. Such interesting and valuable studies were mainly reported by the groups of Coleman^29^^,^^30^ and Bertini-Scozzafava^27^^,^^28^, which reinforced the earlier conclusions^3–5^ that the anion CAIs coordinate to the metal ion in the enzyme active site in a tetrahedral or more rarely, a trigonal bipyramidal geometry^26–30^, as shown schematically in Figure 2 for some anion inhibitors, again using hCA II as model enzyme and high-resolution X-ray crystallography^31–33^.

**Figure 3.**
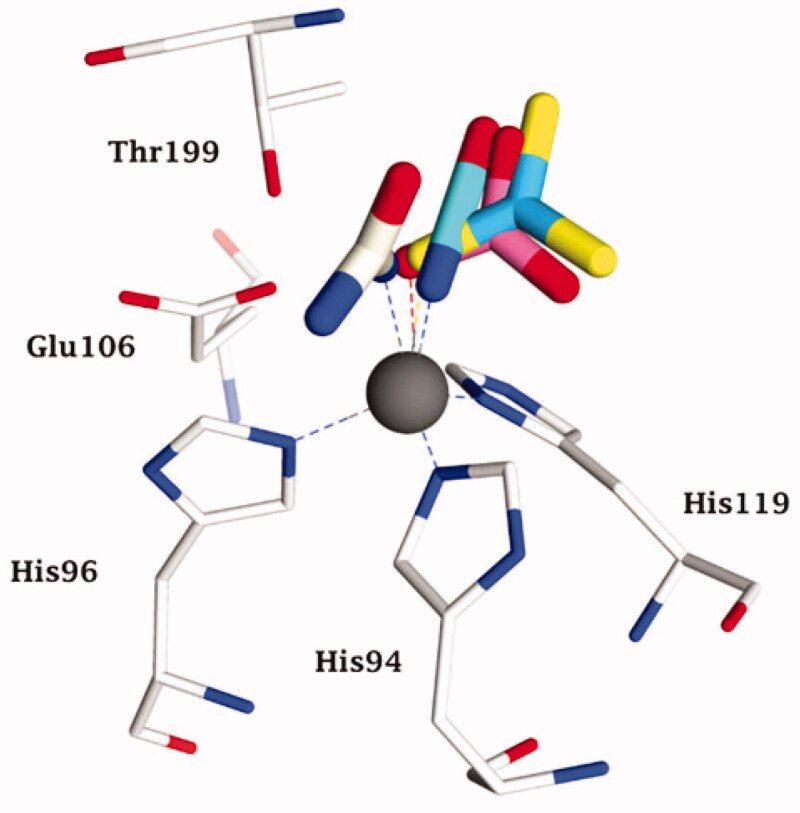
Representation of the superimposed hCA II adducted to cyanate (cyan, pdb 4E5Q^31^) bicarbonate (pink, pdb 2VVB^24^) urea (as anion) (white, pdb 1BV3 [39]), and trithiocarbonate (light blue, pdb 3K7K^40^) The zinc ion (grey sphere), its protein ligands (His94, 96 and 119) and gate-keeping residues (Thr199, Glu106) are also shown.

Clearly based on the data in Figure 2, all these anion inhibitors from significantly different chemical classes (e.g. halides, azide, cyanate and even oxygen, presumably bound as anion or radical anion^33^) act as monodentate ligands to the zinc ion, which is in its usual tetrahedral geometry, with three additional ligands (residues His94, His96 and His119). All these (and other) anions have their closest atom to zinc at a distance of about 2 Å, and most of these anions show a rather low affinity (usually millimolar to micromolar^26^) for this CA isoform. Thus, the zinc remains tetrahedrally coordinated upon inhibition by displacement of a water molecule ligand (or hydroxide) by many different anions. However, in 1993, the results of an X-ray crystallography study performed by the Liljas' group showed that^34^ cyanate and cyanide (well known “metal poisons”) do not inhibit CAs by this metal binding mechanism but instead are bound at a non-coordinating distance, presumably in the same location that the substrate CO_2_ binds (n.b. the CO_2_ binding mode was not precisely known at that time). Liljas presented these results in 1992 at an international bioinorganic chemistry conference in Florence prior to their publication in the following year^34^, in which many scientists active in the CA field participated. This discovery was considered by many to be a turning point in our understanding of CA inhibition mechanisms. However, these surprising findings presented by Liljas were difficult to rationalise, especially considering the fact that Bertini and Scozzafava’s groups, who continued the early research lines pioneered by Lindskog and Coleman, mentioned above^5^^,^^29^, presented highly convincing evidence that cyanate and cyanide coordinate to the metal ion (zinc or cobalt) in the CA active site based on data from electronic spectroscopy, EPR and NMR methods^27^^,^^28^. One of us (CTS) proposed at the 1992 meeting that cyanate and presumably also cyanide might instead be hydrated by the CA enzyme given that these anions have a shape that is rather similar to that of the substrate CO_2_, hypothesis which has been validated in an interesting computational study by Merz and Banci’s groups^35^. Although the eventual experimental validation that CAs can hydrate cyanate and cyanide was more arduous than initially thought, ultimately Maren’s group and one of our groups (CTS) demonstrated that this was definitively the case^36^. Maren’s laboratory was highly skilled in measuring inhibition constants of anions, including halides, cyanate, cyanide, etc^37^^,^^38^. as well as sulphonamides, considering that he was a co-discoverer of several sulphonamide CAIs that have been used clinically for decades as well as the developer of two assay methods based on the CO_2_ hydration reaction^2^. Maren’s group measured the liberation of ammonia from carbamate formed by CA-catalyzed cyanate hydrolysis^36^. Although the ammonia formation was measured, the amount of ammonia was near the limits of detection for the method that was used and no clear-cut conclusions could be drawn. However, we proposed that cyanate once hydrolysed to carbamate within the CA active site may act as a suicide inhibitor, remaining strongly coordinated to the zinc ion^36^, which might also explain the uncommon crystallographic results reported in ref.^34^ The controversy was resolved years later by McKenna’s group^31^ who reported a high-resolution X-ray crystal structure of hCA II with cyanate bound (Figure 2). Cyanate is directly coordinated to the metal ion just like most other anion inhibitors that have been investigated^31^. Although this definitive data was published in 2014, which should have “put the debate to rest”, the egregiously incorrect conclusion from the Liljas paper of 1993^34^ has not yet been rectified by a retraction or correction. Furthermore, the protein database files of the two adducts to hCA II with cyanate and cyanide from Liljas’s group have not been deposited in the Protein Database.

However, this research line ultimately produced some interesting results. Cyanamide (H_2_N-CN or NH = C=NH, as possible tautomers) is another molecule with a similar linear shape with respect to the *sp* hybridised carbon atom as cyanate and CO_2_. Briganti et al.^39^ investigated the role of cyanamide as either a substrate or inhibitor for CAs by using kinetic, electronic spectroscopic and X-ray crystallographic techniques. Again, and to the great surprise, it has been possible to demonstrate that cyanamide is initially acting as a weak inhibitor, which can then undergo a hydration reaction within the enzyme active site resulting in the formation of urea (more precisely the ureate anion), which can remain coordinated to the metal ion, as shown in Figure 3, in which other structurally-related anions, such as cyanate^31^, carbonate^24^ and trithiocarbonate^40^ are also shown superimposed.

**Figure 4.**
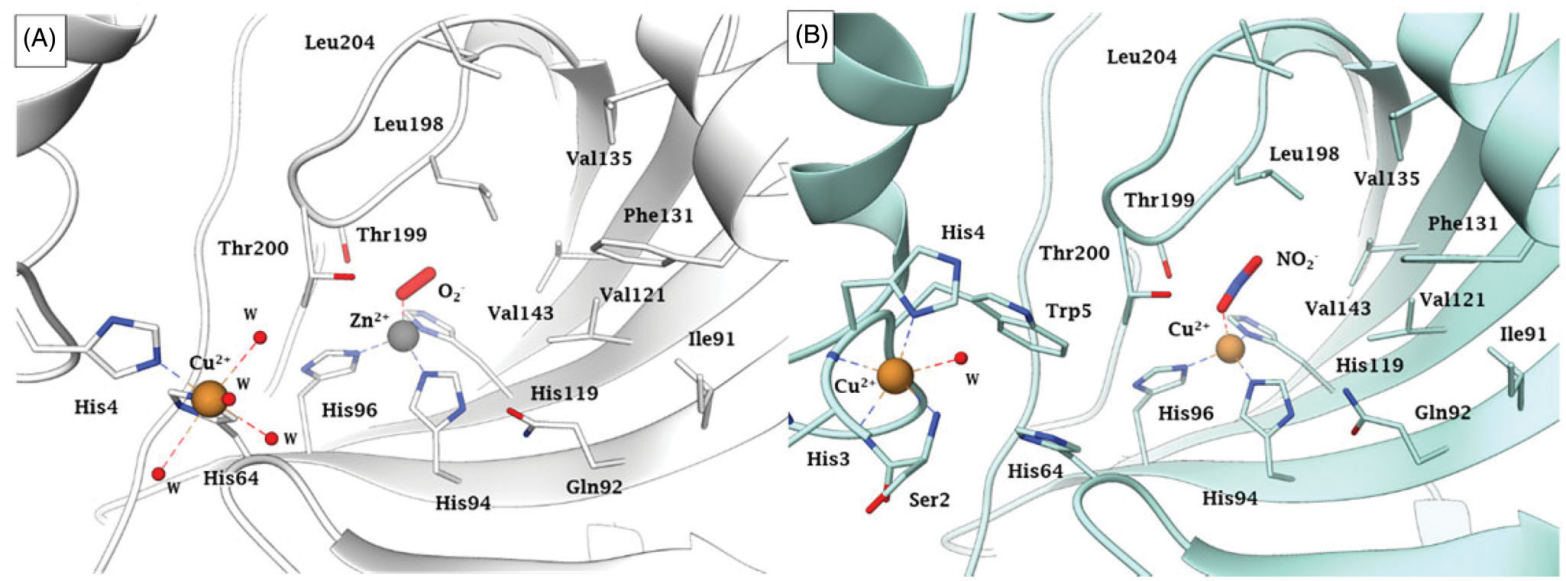
Representation of the binding of A) O_2_^−^ to a Zn^2+^/Cu^2+^ hCA II (pdb 5EOI^33^) and B) nitrite to Cu^2+^ substituted Cu_2_-hCA II (pdb 6PDV^43^) Distances of the closest atom of the anion to the metal ion are: Zn-O (in the O_2_ adduct) of 1.88 Å; Cu-O (nitrite adduct) of 2.14 Å.

Cyanamide (unlike cyanate) was thus proven to be a new CA substrate, which is not that surprising considering that it is isosteric and isoelectronic with CO_2_^39^_._ However, the most interesting result was probably that urea could not be displaced from the inhibited enzyme even by using highly effective, low nanomolar sulphonamide inhibitors, which demonstrated that ureate is probably one of the most effective CAIs known to date^39^. It should also be noted that urea (neutral molecule) is a very ineffective inhibitor (no K_I_ was measured up to 100 mM) probably because it cannot be readily deprotonated in aqueous solutions for enzyme measurement assays^39^. Thus, only the deprotonated form, which is formed *in situ* through the suicide inhibition effects of cyanamide acts as a CAI, which is in fact the situation we expected (but could not initially confirm) for cyanate. As seen in Figure 3, cyanate, carbonate and trithiocarbonate (all zinc binders) do not completely overlap in the X-ray crystal structures but they bind in a similar fashion, being monodentate ligands of the Zn(II) ion, which is in its stable tetrahedral geometry^24^^,^^31^^,^^40^. The same geometry was observed also for the ureate adduct (Figure 3) and it was also bound in a monodentate fashion like many other small anions that have been reported to date. However, the ureate is tilted and has a very different orientation towards the gate-keeping residues Glu106-Thr199^39^. In a subsequent study^41^, by using cryo-crystallography, it has been possible to “freeze” and observe the formation of the intermediates in the CA-catalyzed cyanamide hydration reaction, which revealed a new proposed reaction mechanism for this hydrolytic process.

Apart the active site metal ion, hCA II has an additional metal binding site, at its entrance^42^. Copper(II) binds rather efficiently in this additional site, being coordinated by His64, His4 and several water molecules^33^^,^^42^. This form of the enzyme, with Zn(II) at its active site and Cu(II) at the secondary site is referred to as Zn/Cu-hCA II, in contrast to the enzyme which has zinc substituted by copper also within the catalytic centre (Cu_2_-hCA II)^43^. Recently, McKenna’s group proposed that the Cu_2_-hCA II also has nitrite reductase activity, with nitrite being initially coordinated (as a bidentate ligand) to the active site copper ion (Figure 4(B)). In contrast, as mentioned also above, the Zn/Cu-hCA II was observed with an O_2_ molecule coordinated to zinc (Figure 4(B)), which most probably is a superoxide anion (or anion radical), as suggested by computational techniques^33^. A major overall conclusion is that all serious studies performed to date on hCA II demonstrate that anions are coordinated to the metal ion in the enzyme active site.

## 3. Sulphonamides and other zinc binders, the tail approach

**Chart 1.**
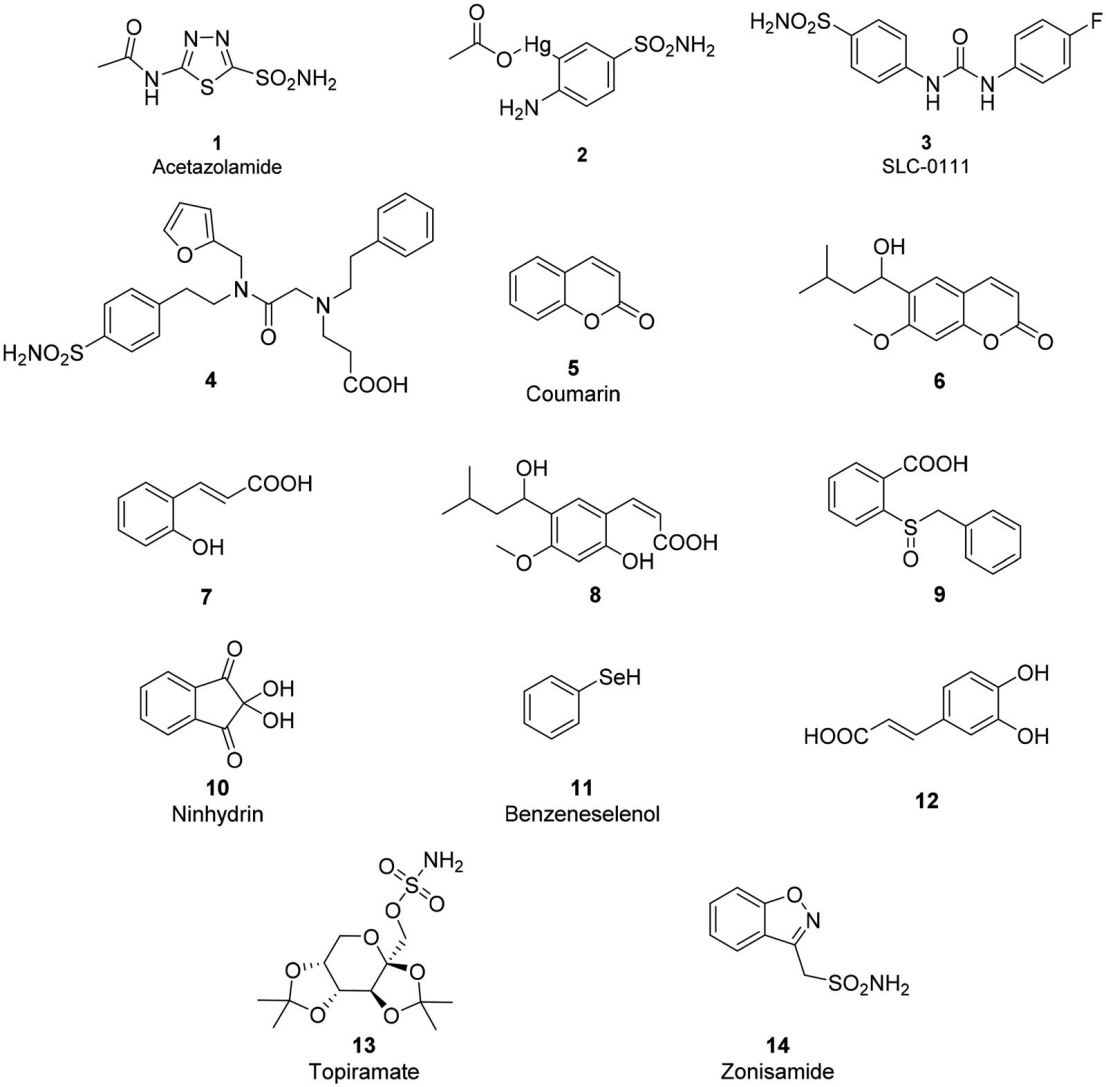
Structure of the compounds **1–14** discussed in this review.

Primary sulphonamides^44–46^ were known for decades^8–10^ to potently inhibit CAs by binding as RSO_2_NH^-^ anions to the Zn(II) ion within the enzyme active site, as shown by using various spectroscopic techniques^5^^,^^12^^,^^13^^,^^30^ as well as a crystallographic study from Liljas’ group, in which acetazolamide **1**, and 3-acetoxymercuri-4-aminobenzenesulfonamide **2** (Chart 1), and the anion thiocyanate, were crystallised in complexes with hCA II^47^. As for the anions mentioned in the preceding paragraphs, the sulphonamide inhibitors substituted the water/hydroxide ion acting as the fourth ligand to the tetrahedrally coordinated zinc site in a monodentate coordination with the metal ion through the deprotonated nitrogen atom of the sulphonamide moiety, resulting in a favourable hydrogen bond with Thr199, a conserved residue in most α-CAs. In contrast, thiocyanate binding was reported to result in a trigonal-bipyramidal coordination of the zinc centre because SCN^-^ added to the coordination sphere of the non-inhibited enzyme^47^. Since then, hundreds of sulphonamides were crystallised in complexes with various CA isoforms and in all of them the sulphonamide zinc-binding group (ZBG) participates in the same interactions as mentioned above for acetazolamide (reviewed in refs.^14^^,^^48^).

Although acetazolamide and other first generation sulphonamide CAIs (such as methazolamide, ethoxzolamide, dichlorophenamide, etc.; refer to refs.^11^^,^^12^^,^^14^ for their structures) are potent, low nanomolar CAIs, they are non-selective inhibitors^11^, strongly inhibiting most hCA isoforms that are known to date (12 catalytically active isoforms that have been reported, hCA I-VA, VB, VI, VII, IX, XII-XIV^11^^,^^12^^,^^14^) As a consequence, the first- and even second-generation inhibitors used clinically resulted in a range of documented side effects connected to the inhibition of off-target isoforms^48–57^.

**Figure 5.**
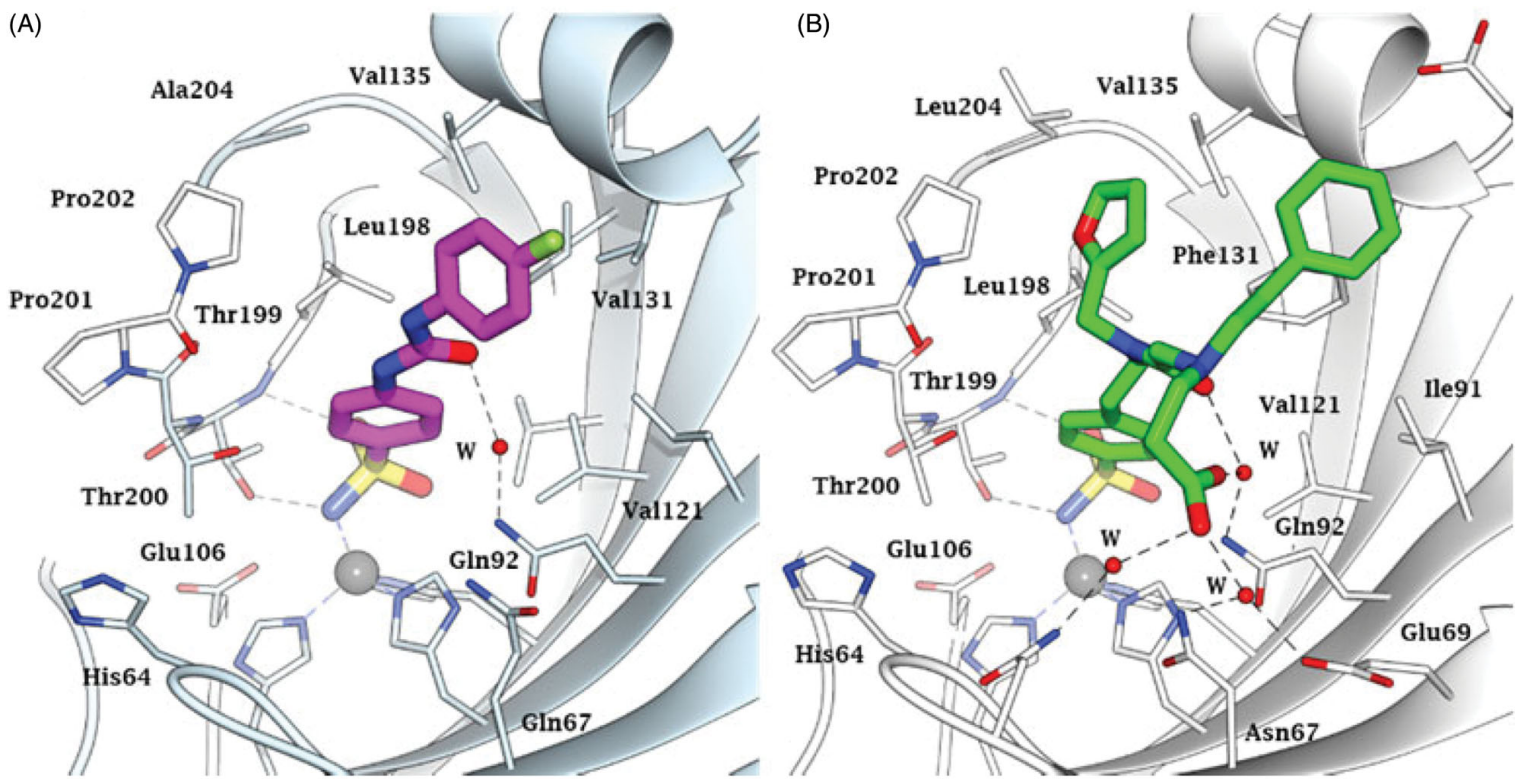
Active site view of A) hCA IX catalytic domain adducted to SLC-0111 (magenta)^74^ and B) hCA II adducted to the three tailed inhibitor **4** (in green).^71^ H-bonds are represented as black dashed lines. The Zn(II) ion (grey sphere) and some important amino acid residues involved in the binding of inhibitors are shown.

For such reasons, designing isoform-selective CAIs became the main issue in the revival of the CA field in the mid to late 90 s for obtaining compounds with an improved safety profile and efficacy compared to the inhibitors available at that time^58–60^. The tail approach has thus emerged in that period as an innovative modality for developing CA-selective inhibitors^61^. The idea is very simple: the attachment of moieties that may induce the desired physico-chemical properties (e.g. enhanced hydro- or liposolubility) to scaffolds of simple aromatic/heterocyclic sulphonamides (e.g. sulphanilamide, metanilamide, orthanilamide, and their derivatives; 5-amino-1,3,4-thiadiazole-2-sulphonamide, 4-methyl-5-imino-1,3,4-thiadiazoline-2-sulphonamide, and many other simple sulphonamides etc.) might lead to interactions with the external part of the CA active site, and not only with residues near the zinc catalytic centre that are the most highly conserved in all hCA isoforms. Indeed, the region at the entrance of the active site cavity is the most variable between the different human isoforms^61^. Detailed kinetic studies were thereafter performed on over 10,000 different sulphonamide derivatives^62–71^, which were synthesised and investigated in our laboratories and an additional 15,000 derivatives were received from academic groups and pharmaceutical companies in collaboration with the Florence group. Crystallography in complex with various isoforms was done on several hundred such derivatives. This type of research demonstrated that the hypothesis was correct and that it is possible to design sulphonamide CAIs that are selective for each isoform of interest by tailoring the dimensions (length, bulkiness, etc.) as well as the chemical nature of the various tails^61–71^. This research led to the discovery of many new sulphonamide, sulfamate and sulfamide classes, including the ureido-benzene-sulphonamides^70^^,^^72^ as well as derivatives possessing more than one tail attached to the scaffold, such as dual-tailed or tri-tailed CAIs^73^^,^^74^. Of the many compounds discovered in this way includes SLC-0111, compound **3**, which is a CA IX/XII-selective inhibitor that is now in clinical trials as an antitumor/antimetastatic agent (see discussion later in the text). Another notable example is the three-tailed sulphonamide **4** (Chart 1).^71^ Both SLC-0111 and sulphonamide **4** strongly inhibit and bind to hCA IX (Figure 5)^71,74^.

**Figure 6.**
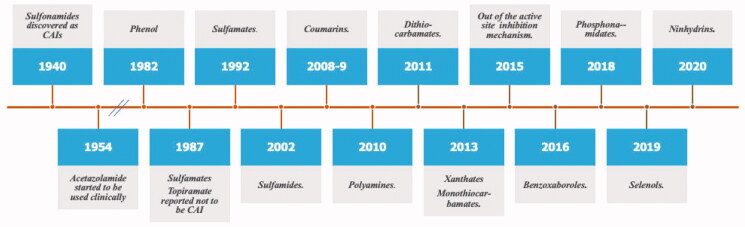
Historical overview on the discovery of the main chemotypes with CA inhibitory activity.

The tail approach was virtually the only strategy used to obtain CAIs from the moment it was disclosed, and it has been adopted by research groups all over the world^71^^,^^75–91^. For space reasons only a limited number of such research was cited here, as the field was recently reviewed^14^^,^^48^. The advantage of the tail approach over other strategies to obtain isoform-selective CAIs are the facility of the syntheses, the versatility and possibility to introduce a variety of tails/scaffolds with different chemical properties, and thus to generate chemical diversity. As a consequence, these procedures were extended to many other classes of CAIs, not only to the sulphonamides and their isosteres (sulfamides/sulfamates, which are not discussed here in details; see refs.^14^^,^^48^ for recent reviews). These new chemotypes will be discussed in the next paragraph, together with some new CA inhibition mechanisms which emerged more recently.

## 4. Other chemotypes with CA inhibitory power

By comparing the sulphur-based, carbon-based^45^ and phosphorus-based ZBGs, a range of novel classes of non-sulphonamide CAIs which interact with the zinc ion were reported, including phosphonamidates^91^, dithiocarbamates^92^ and their derivatives (monothiocarbamates^93^, xanthates and trithiocarbonates^94^) selenols^95^^,^^96^, carboxylates^97^, hydroxamates^98^, benzoxaboroles^99–101^, and carbamates^102^. In addition, other classes of inhibitors that have been developed which exhibit a different inhibition mechanism compared to the zinc binders discussed above. It should be noted that many of the new chemotypes bind further away from the metal ion, interacting both with the hydrophobic and hydrophilic halves of the CA active site^14^^,^^48^. These new inhibition mechanisms include:

- CAIs that anchor to the zinc-coordinated water molecule^103^^,^^104^. Polyamines, such as spermine and its derivatives^103^, the simple phenol^104^ and many of its derivatives^105–108^ together with polyphenols^109^ inhibit CAs in this way. This inhibition mechanism is also shared by the sulfocoumarins and their derivatives, as demonstrated by Zalubovskis group^110–113^ using kinetic and crystallographic studies. These classes of highly innovative CAIs were developed by the Riga group^110–113^ starting from coumarins and their derivatives as a class of inhibitors with a totally new inhibition mechanism, which has been unravelled by some of us^114–124^. Thioxocoumarins are the only exception, as they bind similar to the phenols, anchoring with their exocyclic sulphur atom to the zinc coordinated water molecule^114^.
- Coumarins and many of their derivatives such as mono- and dithio-, as well as seleno/tellurium-coumarins but also many other structurally related compounds^115–124^ were shown to possess a very particular inhibition mechanism. This was demonstrated for the simple coumarin **5** and one of its derivative (compound **6**), a natural product isolated from the Australian plant *Leionema ellipticum*^115^^,^^116^. The lactone ring of the coumarin is hydrolysed by the esterase activity of CA, leading to the formation of *cis*- or *trans*-2-hydroxycinnamic acid derivatives, of types **7** and **8** (Chart 1), which thereafter bind far from the zinc ion, at the entrance of the active site cavity (the region which is more variable among the different human isoforms, as mentioned above). As a consequence, coumarins and their derivatives behave as pro-drug inhibitors, but also show a very high selectivity for many CA isoforms, depending on the substitution pattern on their bicyclic ring system^115–124^. Many representatives belonging to this family of CAIs in fact possess extremely good selectivity ratios for inhibiting isoforms of interest (e.g. CA IX and XII) versus highly distributed, “house-keeping” off-target isoforms, such as CA I and II^115–124^.
- Probably one of the strangest CA inhibition mechanism is the one reported by De Simone’s and Carradori’s group for a benzoic acid derivative (compound **9**) which binds outside the active site cavity, blocking the His64 residue (the proton shuttle residue) in its *out* conformation^125^. Again, X-ray crystallography was crucial for demonstrating this highly interesting CA inhibition mechanism.

**Figure 7.**
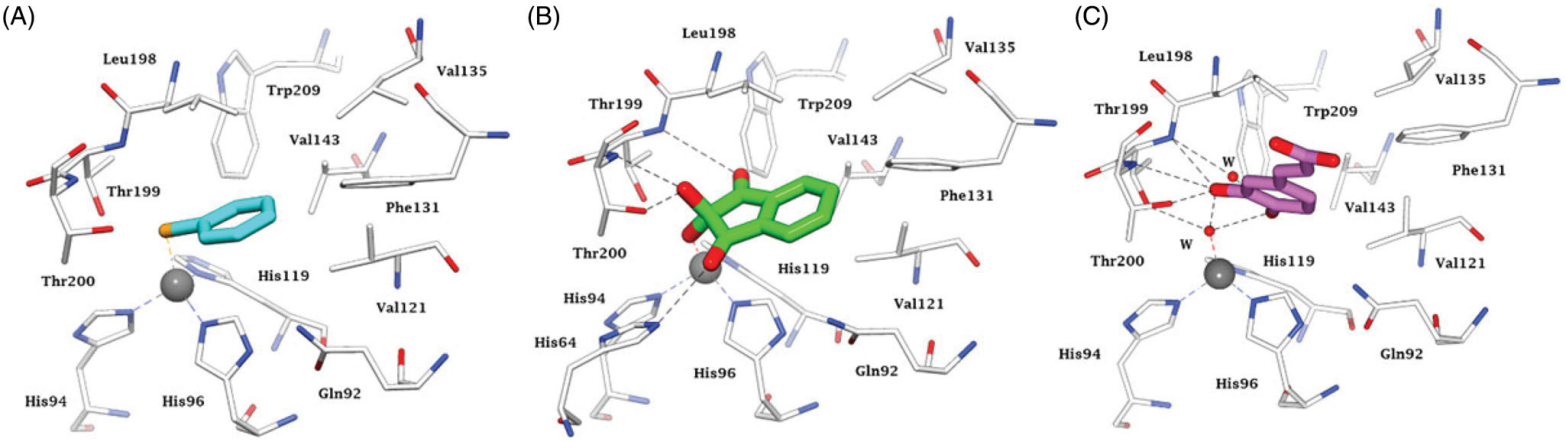
Active site view of hCA II complexed to A) selenol **11** (pdb 6hX5) ^96^, B) ninhydrin **10** (predicted *in silico*)^127^ and C) catechol derivative **12** (pdb 6YRI) ^128^. H-bonds are represented as black dashed lines. The Zn(II) ion (grey sphere) and residues involved in its coordination and some active site residues near the binding of inhibitors are shown in CPK colours. Water molecules are shown as red spheres.

An overview of the main new chemotypes acting as CAIs discovered up until now is shown in Figure 6, in which the “fresh” discovery of the ninhydrins is also mentioned^126^. Indeed, ninhydrins, such as compound **10**, possess a *gem*-diol stable functionality which has been shown by means of kinetic and computational techniques to coordinate to the metal ion from the enzyme active site^126^. Furthermore, Figure 7 shows the X-ray crystal structures of some of the new chemotypes detected in the last period, such as the selenol **11**^95^ (Figure 7(A)), unsubstituted ninhydrin **10**^126^ (Figure 7(B)) and a catechol derivative **12**^127^ (Figure 7(C)) which has been recently reported to bind to hCA II by a new inhibition mechanism. Like many phenols, the catechol **12** is anchored to the zinc-coordinated water molecule, but being a diphenol, the second OH moiety additionally binds to the “deep water” molecule in the active site. As mentioned above, selenol **11** and the ninhydrin **10** (Chart 1) directly coordinate to the zinc ion in the enzyme active site (Figure 7). Many of these inhibition mechanisms were thoroughly reviewed in ref.^128^.

**Figure 8.**
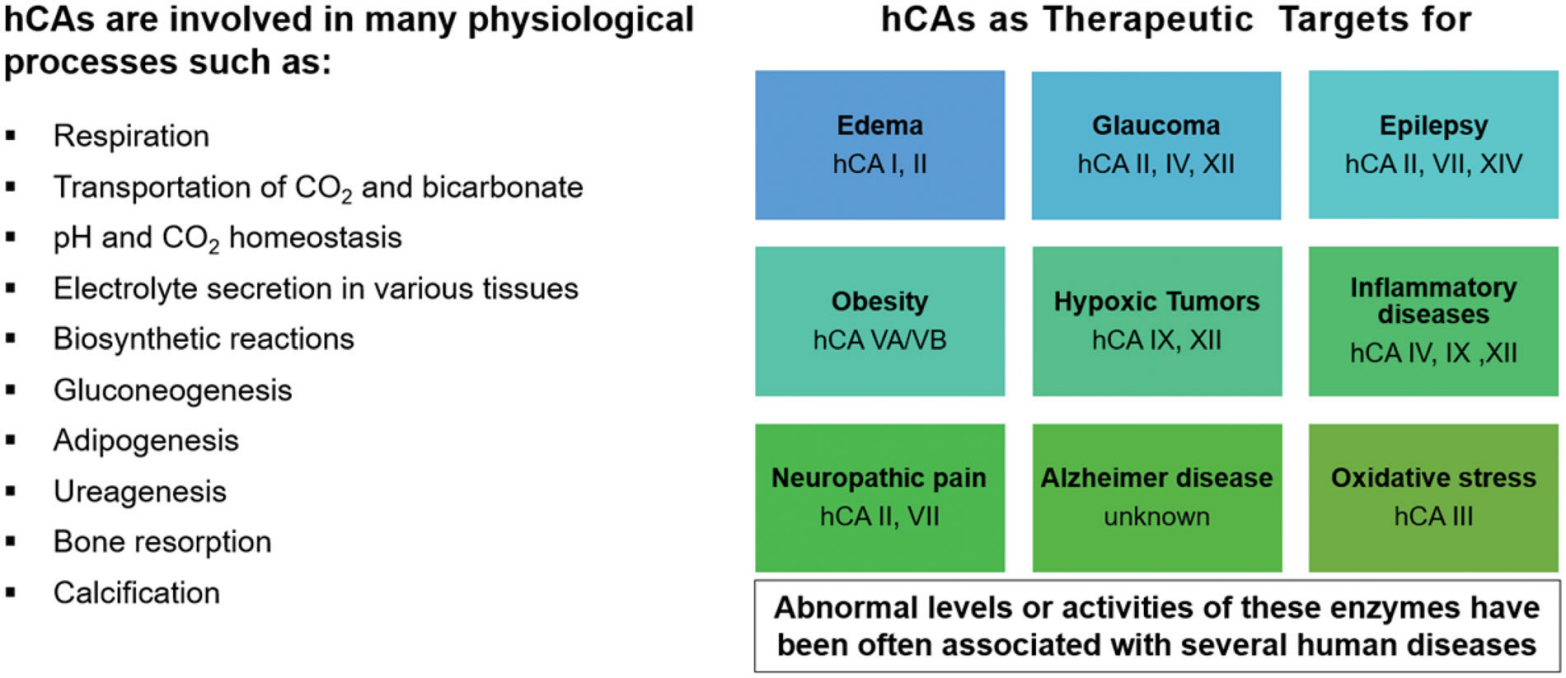
hCAs as drug targets^11^^,^^14^^,^^51–57^. From edoema and glaucoma, to obesity, neuropathic pain, hypoxic cancers, cerebrovascular diseases and oxidative stress, many isoforms are involved in diverse pathologies for which isoform-selective inhibitors showed a relevant potential to be translated to clinical entities.

## 5. New applications of the CAIs

For the last 65 years the CAIs were used as diuretics^2^^,^^51^^,^^53^^,^^55^ and anti-glaucoma agents^2^^,^^56–59^, and they still have a firm place for the management of these conditions^53^^,^^57^. However, in the last decade, several other highly relevant applications emerged, probably due to the availability of much more isoform-selective inhibitors. They include the use of CAIs such as acetazolamide **1**, topiramate **13** and zonisamide **14** as antiepileptics^129^^,^^130^ and anti-obesity agents^54^^,^^131^^,^^132^, targeting diverse isoforms (CA II and VII for the antiepileptic use, and CA VA/VB as antiobesity agents) ^129–132^. In fact, as seen from Figure 8, most of the CA isoforms, alone (or in combinations of few of them) possess therapeutic/pharmacological applications.

In fact, nowadays the CAIs were proposed to be used or are already used for the management of the following pathologies, in addition to the classical ones mentioned above: (i) treatment of idiopathic intracranial hypertension, by targeting hCA I and II but presumably also brain/cerebrospinal fluid isoforms^133^; (ii) cerebral ischemia/diabetic cerebrovascular pathologies (the targeted isoforms seem to be hCA IX and XII, possibly also hCA VA)^134^; (iii) neuropathic pain, by targeting the bran/peripheral nervous system isoforms hCA II and VII^135^^,^^136^; (iv) inflammation/arthritis, by targeting hCA IX/XII and possibly hCA IV^137–140^; (v) hypoxic tumours, by targeting the two isoforms discovered to be associated with tumours, hCA IX^141^ and hCA XII^142^. This is in fact the field in which the highest number of studies have been published in the last decades^143–155^. Most of the proof-of-concept studies which validated the various isoforms for the management of such widespread diseases were conducted either in Florence or by collaborations with well-known groups worldwide, which used the compounds developed in Florence for these studies. Highly important for the success of these investigations was the fact that the X-ray crystal structures of almost all hCA isoforms (except hCA VB, X and XI) were published in the last 15 years^156–161^. This allowed for highly focussed drug design campaigns in order to obtain isoform-selective compounds for all these enzymes, and again using the tail approach^61^ and a variety of newly discovered chemotypes, discussed above, were essential for the success of these efforts.

## 6. CA activators and their applications

The carbonic anhydrase activators (CAAs) have been the focus of significant controversy for a long time up until 1997 when the first X-ray crystal structure of a complex of an activator (histamine) bound to hCA II was reported and the activation mechanism was revealed in molecular level detail^162^. The activator, which was observed to be directly bound within the enzyme active site, promotes the formation of an enzyme – activator complex, in which the proton shuttling moieties present in the activator participate in the rate-determining step of the catalytic cycle, i.e. the transfer of a proton from the zinc-coordinated water to the external reaction medium, similar to the natural proton shuttle, which is residue His64 (in many CA isoforms), as mentioned above^162^^,^^163^. In such enzyme-activator complexes, the proton transfer is intramolecular, being more efficient compared to the intermolecular transfer to buffer molecules, which are not bound within the enzyme cavity^162^^,^^163^. Many X-ray crystal structures with amines and amino acid activators were reported thereafter, in addition to histamine^162^, including L- and D-His bound to hCA II and hCA I, L- and D-Phe, D-Trp, L-adrenaline and pyridinium derivatives of histamine bound to hCA II, which confirmed this general CA activation mechanism outlined above^164–167^. The thirteen catalytically active mammalian CAs (e.g. CA I-VA, VB, VI, VII, IX, XII-XV) were also investigated for their interaction with a rather large library of amino acids and amines^168^^,^^169^, whereas several drug design studies have also been performed^170–174^. Why are these studies relevant? In the last few years it has been demonstrated that CAAs of the amino acid type (e.g. D-Phe) can enhance memory and learning, which is antagonised by the simultaneous administration of a CAI of the sulphonamide type (e.g. acetazolamide) ^175^. A recent study showed that administration of the D-Phe to rats rapidly activated the extracellular signal-regulated kinase (ERK) pathways, which are involved in critical steps of memory formation, both in the cortex and the hippocampus, two brain areas crucially involved in memory processing^175^^,^^176^. Even more recently, the same groups (including the Florence team) demonstrated that CAAs play a crucial role in extinction of contextual fear memory^177^, opening the way towards the validation of CAs as new targets for improving cognition, but also in therapeutic areas, such as phobias, obsessive-compulsive disorder, generalised anxiety, and post-traumatic stress disorders, for which few efficient therapies are available to date^175–177^.

## 7. Discussion and conclusions

The drug design landscape for modulators of CA activity, both inhibitors and activators, appears to be a dynamic and healthy field, in which many relevant advances have been achieved in the last several decades through continuous contributions from many research groups, as outlined above. However, recently an event disturbed this situation. In an egregious polemic from Jonsson and Liljas^178^, the measurements of some CA inhibition data were queried, in addition to some other aspects that will be discussed later in this article. This action was based on some typographical errors found in Supplementary Information from 5 papers from our group (among the > 1600 that have been published). We have replied to the above-mentioned article^178^ in an Editorial published in the same journal^179^ as well as two additional review articles^153^^,^^180^. Jonsson and Liljas replied to our editorial^179^ with a new comment published recently^181^. Furthermore, Liljas “promoted” their initial article^178^ by writing a denigrating and arrogant letter to the president of the University of Florence about one of us (CTS), stating that he is, “*bothered about how long his* [Supuran’s] *poorly performed science could continue without serious criticism*”, and “*I followed Supuran's crystallographic investigations with horror*”, but no technical comments regarding our presumed crystallographic errors were mentioned in this letter or in their papers, although Liljas himself is a crystallographer. We worked in this field in collaboration with well-established crystallographers from several laboratories in Italy, Germany, USA, Australia and Singapore over the years^178^. Liljas continued, “*the measurements are so poorly done that an undergraduate student reporting these in his works should have failed*” and “*we tried to publish our manuscript in the journals where Supuran has published his work but failed. This covers a good number of journals*”, meaning probably that serious journals did not take into consideration his poorly formulated polemic. Liljas ends his letter to the rector: “*The work by Supuran that we criticise needs to be repeated. There may be really excellent inhibitors among the hundreds of compounds that he has studied, but his analysis does not give a trustworthy account. It is important that the field and his co-workers become fully aware of the quality of his analyses and to know that much has to be done again*”. In other words, this scientist who published completely erroneous conclusions based on crystallography data as shown here for the cyanate and cyanide examples^34^ and elsewhere by others^31^ and who has not withdrawn or corrected these published results after 27 years, finds it insufficient to publish his conceptually erroneous polemic^178^, but also wants to defame our group to our institution. We have replied to the technical issues raised by Jonsson and Liljas, as mentioned above. In their comment on our reply^180^ they indicated they were “*happy by our prompt response*” and that some clarifications were achieved^181^, however now their criticism relating to the measurement of inhibition constants was extended to the entire medicinal chemistry community, not only to our group, stating that the “*original article was not intended to raise specific concerns regarding just one research group but rather was intended to raise concerns regarding medicinal chemistry publications in general, and we are glad that Prof. Supuran agrees that there is room for improvement!*” CTS hardly agrees with anything from their analysis. Such a strong and prejudicial opinion from someone without experience in the medicinal chemistry field is disappointing. Notably, the “best practice” examples for the measurement of K_I_s that Jonsson and Liljas provide in their reply^181^ are the highly controversial articles from Johnson & Johnson regarding topiramate **13**^182–185^. Indeed, topiramate was initially reported to not be a CAI^182^, then a millimolar inhibitor^183^, then a micromolar inhibitor^184^ until finally it was established to be a sub-micromolar inhibitor^185^ by the same group of Johnson & Johnson who initially discovered the compound. We have reported the X-ray crystal structure of this important drug bound to hCA II and demonstrated that it is a low nanomolar inhibitor for many CA isoforms^186^. There are also other unusual comments from Jonsson and Liljas^181^ regarding the native mass spectrometry measurements. Presumably, the main issue with this method is that it can be used to measure dissociation constants that are in very good agreement with the stopped flow method used in Florence, in addition to other methods such as isothermal titration calorimetry (ITC) and differential scanning calorimetry (DSC)^187^^,^^188^.

As noted above, there are no original contributions in the literature from Jonsson and/or Liljas regarding the measurement of inhibition constants (for CAs or any other enzyme) nor for any drug design studies targeting CAs or other druggable proteins. Jonsson is a biochemist who published several papers in the CA field and Liljas was a crystallographer (now retired for some time), who, as we mentioned earlier, contributed some important X-ray crystallographic studies in the 70 s and 80 s on CAs and then some questionable CA data in the 90 s (as discussed above). It should be noted that there is no analysis at all of our crystallographic work in the two papers by Jonsson and Liljas^178^^,^^181^ or in the letter to the rector, from more than 250 papers that we have published in that field, as mentioned here and elsewhere^12^^,^^14^. However, the polemic by Jonsson and Liljas^178^ has been supported by another scientist, Matulis, based on his recent reference to this work^189^.

Matulis entered the CA field in 2005 when he published his first paper with the Johnson & Johnson group^190^, on a fluorescent thermal shift assay (FTSA) for measuring CA inhibition. In this method, the extent of protein unfolding is monitored as a function to temperature when heated, alone and in the presence of inhibitors, providing a way to measure the affinity of inhibitors as well as some thermodynamic parameters. This method requires high concentrations of enzyme (around 5–10 µM), it uses a very high concentration (50 µM) of a fluorescent dye, ANS (8-anilino-1-naphthalene-sulphonate) as well as a 50 mM phosphate buffer^190^. We and others have demonstrated that sulphonates can be strong inhibitors of the CAs^110^, and phosphate is also an anion CA inhibitor^26^. Just recently one of us (AA) retested phosphate as an inhibitor of hCA I and II at a concentration of 10 nM to 100 mM by the stopped-flow assay. At 50 mM, an inhibition of 13.0% of the activity of hCA I and 19.1% of the activity of hCA II was measured (unpublished results from our laboratories). Thus, the competition of the buffer, dye and inhibitor for binding to the metal ion (or elsewhere in the CA active site), coupled with the large concentrations of enzyme that is required results in this method providing information of limited usefulness regarding CA activity. FTSA was however extensively used in the subsequent Matulis papers, in combination with ITC, which as mentioned by Linkuviene et al. is one of the “most robust techniques” for measuring interactions between proteins and small molecule ligands in drug design^191^. ITC also uses high concentrations of enzymes in the assay system (6–20 µM), and as mentioned in the Matulis papers the buffer is again 50 mM phosphate plus 100 mM NaCl. In addition to phosphate, chloride is also a CAI that binds to the metal ion active site^26^. Chloride is even more inhibitory than phosphate, as documented earlier by many groups. The K_I_ of this anion is 6 mM against hCA I, 200 mM against hCA II, 1.8 mM against hCA VII, 138 mM against mCA XIII (m = murine enzyme), 33 mM against hCA IX and 0.8 mM against hCA XIV^192^. Thus, it is unclear how robust the inhibition data is in these ITC studies when such high concentrations of CA anion inhibitors are being used.

In the subsequent papers from Matulis’ group^193–198^, all of which were inspired by the tail approach (see above), reported by us, a combination of several CA inhibition assays were performed, including the FTSA method^190^ and the ITC assay mentioned above, in addition to the stopped flow assay, which members of the Matulis group learned in 2014 in Florence at the request of Matulis (and we accepted them and taught them the CO_2_ hydrase assay developed originally by Khalifah^199^ which has been used in Florence since 2001). Notably, the stopped flow assay uses nanomolar concentrations of CA in the system and buffers such as Hepes or Tris at 10–20 mM (which do not interfere with the metal ion), phenol red (at a concentration of 0.2 mM as an indicator) as well as low concentrations of salts to maintain a constant ionic strength (typically 20 mM NaClO_4_, which is not inhibitory to most CAs^26^^,^^192^) Needless to say, all the methods used by Matulis gave very different results, and it is often difficult to understand which of them should be considered for evaluating the inhibitors from his laboratory.

Thus, the next step of Matulis’ research was to investigate the so called “intrinsic” parameters for characterising the binding of inhibitors to various CA isoforms (there is a series of 24 papers in which “intrinsic” is the keyword, repeated with great insistence, but we will consider only the one in which the method is presented in detail^200^) The main issue with the measurements of CA inhibition from all other laboratories around the world and in all historical periods, is according to Matulis that “many protein-ligand binding reactions are linked to protonation/deprotonation reactions or various conformational changes”, which were not considered by other researchers. N.b., conformational changes are not considered in this “intrinsic” parameter model either. Thus, the measurement of the energetic contributions for enzyme-inhibitor complex formation is considered to be correct only by considering additional contributions such as protonation/deprotonation reactions of the ligand and the protein. In this way, the observed parameter (e.g. K_I_, ΔH, ΔG, etc.) must be “corrected” to obtain the “intrinsic” value (K_I_^in^) by dividing the observed parameter (K_I_^obs^) by the product of the fraction of deprotonated sulphonamide (f_sulf_) and the fraction of enzyme in the protonated form (f_CA_)^201^. In other words, K_I_^in^ = K_I_^obs^/(f_sulf_ x f_CA_). The theoretical considerations for this “correction” of the observed K_I_ values are based on an old but correct work from Khalifah’s group^202^ in which the binding of imidazole and related compounds to hCA I was investigated. Based on this work Matulis proposes the “intrinsic” parameter model considering that “only the deprotonated form of the sulphonamide binds to the CA active site, and that, the Zn-coordinated hydroxide must be protonated before it can be replaced by the amino group of the sulphonamide”. He cites the above-mentioned Khalifah paper^202^ (based on data for imidazole, which binds in neutral form to the enzyme and does not interact with the metal ion or the water/hydroxide coordinated to the metal ion^202^) to justify his model for sulphonamides, which is erroneous for several reasons which we will detail here:

- Apart the deprotonation of the sulphonamide moiety of the inhibitor and the protonation of the zinc hydroxide species of the enzyme, no hydrogen-bonding or hydrophobic interactions are considered at all in the “intrinsic” model. The Khalifah hypothesis^202^, which is outdated, was appropriate for investigating imidazole and related azoles as CAIs but was never applied to sulphonamides. In fact, in a very important study from Klebe and Cavalli’s groups^203^ it has been demonstrated by using ITC, X-ray crystallography and molecular dynamics (MD) simulations that hydrophobicity is the key factor for the binding kinetics of sulphonamide inhibitors to this enzyme. In fact, by combining the three techniques mentioned above, Klebe and Cavalli’s groups^203^ showed that the protein-sulphonamide association rate (k_on_) dramatically increases with the hydrophobicity of the inhibitor, thus demonstrating the existence of a pre-binding stage in which the enzyme-inhibitor adduct is stabilised by the hydrophobic half of the CA active site^204^. Such hydrophobic interactions are the only ones occurring in the so called “F conformation” of the sulphonamide inhibitor adduct, which in its neutral form (RSO_2_NH_2_) makes a hydrogen bond to the zinc-coordinated water, which is similar to the phenols or polyamines binding to the enzyme by the zinc-anchoring mechanism discussed above^128^. Then the “S state” emerges, which is characterised by geometries that allow hydrogen bonding of the still neutral SO_2_NH_2_ moiety of the inhibitor with Thr199, Thr200, and finally the Zn^2+^-bound hydroxyl ion (not to zinc-water as in the Matulis model). Thus, the sulphonamide approaches hCA II in its neutral state (not deprotonated as in the Matulis model), binds to the hydrophobic patch, and in the end interacts with the Zn^2+^ ion, being deprotonated nearby it, before finally coordinating to the zinc but only if the distance between the terminal NH and the zinc ion is within about 2.50 − 2.75 Å, i.e. the final thermodynamic minimum^203^. These highly relevant data, which are in total contrast with the entire Matulis “intrinsic” theory, were thereafter validated by interesting work from the groups of Klebe^205^^,^^206^ and Whitesides^207–209^, and are supported by our analysis of the hydrophobicity of the CA active site^204^ as well as the report of several classes of inhibitors which anchor to zinc-bound hydroxide/water molecule^128^. Whitesides’ group elegantly demonstrated using ITC, crystallography and MD that the differences in binding between homologous sulphonamide ligands stem from changes in the number and organisation of active site localised water molecules rather than (or perhaps in addition to) the release of structured water from the apposed hydrophobic surfaces^207^. Again, the hydrophobic effects, not at all considered in the Matulis intrinsic model, are the main players in the inhibition process. In a subsequent study^210^ again from the very famous laboratory of Whitesides, it was demonstrated that hydrophobicity is essential also for the binding of anion inhibitors (never investigated by Matulis). It was shown that the binding of the Hofmeister anions is determined by where, and how strongly, they associate with concavities on the surfaces of proteins and how, upon binding, they alter the structure of water within those hydrophobic concavities. It should be stressed here again that in the “intrinsic” method of Matulis, the measurements are done in 50 mM phosphate buffer and 100 mM NaCl, and that these anions are both inhibitory to CAs^26^^,^^192^, as detailed above, because they bind the zinc ion sometimes with a rather relevant affinity, but this phenomenon is never considered properly, and leads to erroneous measurements (apart from the conceptually wrong model used, see above and below).
- Matulis considers that sulphonamides bind to the zinc ion in the CA active site with the sulphonamide group in the deprotonated, negatively charged form, to the water bound form (the so-called acidic form) of the enzyme, which as shown above, is not correct. Although noting that the association rates of sulphonamides to CA are slow, he considers this as “unexpected” considering that association is driven by a strong electrostatic interaction between the negatively charged sulphonamide and the positively charged Zn^2+^. Again, only electrostatic and acid-base equilibria are considered and not any hydrogen bonding and/or hydrophobic contacts, which are as important (or maybe even more important) than the electrostatic interactions in this context. This leads to the discovery of supposedly “kinetic – selective CAIs” ^211^. For decades, the kinetics of association (k_on_) and dissociation (k_off_) of the sulphonamide inhibitors to hCA II were well known^212^^,^^213^. Maren and his group reported the kinetic values for more than 25 sulphonamides directly by measurement of association rate constants (k_on_) and equilibrium constants (K_I_), which were used to measure the dissociation rate constants, k_off_^212^^,^^213^_._ The values for k_on_ ranged from 0.003 to 31 x 10^6^ L^−1^ M^−1^, whereas the k_off_ range was exceedingly low, 0.01–0.05 s^−1^. Thus, the inhibitory activity was entirely influenced by the association rate k_on_ as also detailed by the groups of Klebe, Cavalli and Whitesides^203–210^, which depends primarily on hydrophobic effects. That is, another major error in the analysis of Matulis and colleagues is the lack of considering the hydrophobic effects when analysing the kinetics of association of the sulphonamide inhibitor within the active site. By analysing such low dissociation rates, they reported several ligands to be “kinetic-selective” against certain CA isozymes^211^. Such a proposal is completely at odds with the well-established selectivity of CAIs for 15 CA isoforms from vertebrate organisms that is based on extensive thermodynamic measurements by groups from all over the world. Will the inhibitors reach the thermodynamic equilibrium, or will they “hurry up” to selectively inhibit some isoforms for which the association rates are faster, and in this way provide novel therapeutic applications, as suggested^211^? We leave the answer to this question to the reader.
- Matulis suggested that the observed experimental inhibition constants should not be directly translated into a structure − activity relationship as done by all medicinal chemists, instead proposing that the observed gain in inhibitor potency might be attributed to an increase in p*K*_a_ for the ionisable pharmacophore (presumably deprotonation of the sulphonamide moiety, the only pharmacophore that he has explored) and a corresponding “jump” in the observed affinity instead of to an improvement in ligand-protein interactions. Based on such a model, the explanation he gave for the difference in inhibition between CF_3_SO_2_NH_2_ (a very potent CAI, K_I_ of 2 nM against hCA II, as measured by Maren’s group and confirmed by others^213^) and CH_3_SO_2_NH_2_ (a very weak CAI, K_I_ of 0.1 mM against hCA II, again data from Maren and confirmed by other groups^213^) is dubious. Matulis predicts a 6-fold difference in the K_I_ values between the two sulphonamides based on this theory, whereas the actual measured difference is 5 orders of magnitude. Thus, according to Matulis, only the quantification of intrinsic parameters may allow for the correct understanding of the inhibition mechanism. However, this method has another major issue: it works only with aromatic/heterocyclic primary sulphonamides (the data on aliphatic sulphonamides is illustrated above). All other inhibitor classes (and even a cyclic sulphonamide such as saccharin^214^) cannot be measured with this approach (personal communication from Matulis to CTS). In fact, there are no studies from his group on other types of inhibitors apart primary sulphonamides. As showed here, there is a large variety of other chemotypes with potent CA inhibitory action, and their inhibition effects can be measured and compared well with the sulphonamides by using the stopped-flow CO_2_ hydrase assay queried by Liljas and referred to by Matulis. Note that other groups (Klebe, Whitesides, etc.) which use ITC and other techniques, have not queried the measurements done in our laboratory. So, what is the real problem?

Although we collaborated in the past with Matulis, his behaviour towards some of us should be discussed here. In 2013 at a conference organised by De Simone and one of us (CTS) in Naples, Italy, Matulis was invited as speaker and presented the ITC inhibition method. CTS asked whether the high concentration of sulphonate dye and the sulphonamide inhibitor will all compete for the same binding site and asked whether these effects were considered? There was not an adequate reply from Matulis. Later, during that conference, another author of this paper (RZ) was approached by Matulis who asked him to send him all his compounds that had been tested in Florence, so that he can check if the inhibition data were correct. RZ was shocked by the request and did not send the compounds to his group in Vilnius. The next year, in 2014, as already mentioned here, Matulis asked CTS to host some of his group members in Florence in order to learn the stopped flow assay. CTS agreed, and two students from Matulis’ group spent several months in Florence and learned the assay. In early 2015 after their stopped flow assay was working in Vilnius, Matulis wrote an e-mail to RZ asking him to no longer collaborate with the Florence group because “they do the stopped-flow measurements in an incorrect manner”. RZ replied to Matulis copying CTS and indicated his refusal to collaborate. Only at that point, considering this unethical behaviour, CTS decided to stop any interaction with this colleague.

Returning to the Jonsson and Liljas polemic^178^, although these two colleagues have not made contributions to anticancer drugs or drug design, they questioned the drug candidate, SLC-0111, which is in Phase Ib/II clinical trials for the treatment of advanced, metastatic solid tumours^215^. Jonsson and Liljas state that “it is doubtful that SLC-0111 has the required K_I_ to advance to clinical trials” and that the “commercial interests of pharmaceutical companies and patients may be hurt” ^178^, although the paper in which we describe the compound^72^ is not correctly cited and there are no new data on this compound in their commentary. SLC-0111 was reported by the Supuran group^72^ in efforts that spanned many years during which thousands of compounds were synthesised, assayed and characterised by X-ray crystallography and other biophysical techniques. SLC-0111 belongs to a class of ureidobenzene sulphonamides which were also assessed for their “druggability” criteria, including ADME (Absorption, Distribution, Metabolism, Excretion). Extensive *in vitro* and *in vivo* analysis in appropriate cancer models were then performed in Florence and other laboratories. Although the targets of this compound, CA IX/CA XII, are highly expressed within the hypoxic niches of solid tumours that represents potentially a minor portion of the total tumour cell population, these hypoxic cells have the properties for self-renewal^216–218^, migration/invasion^219–221^, survival in acidic tumour microenvironment^222–227^, and significantly contribute to resistance to chemo-, radio-, and immune-therapies^228–236^. The combination of SLC-0111 and other CAIs with chemotherapy agents^237–239^, immunotherapy^240–242^ and radiotherapy^243^^,^^244^ has been investigated, including SLC-0111, in combination with proton pump inhibitors^236^, antimetabolites^237^, cisplatin^238^, APE1-Ref-1 inhibitors^239^ and histone deacetylase inhibitors^240^, which resulted in excellent synergistic antitumor/antimetastatic effects. Such studies showed a synergistic effect between the CAI and the second antitumor agent, but also the lack of endothelial toxicity^241^, which are highly important and desirable features for a sustained therapeutic response. The extensive studies reported in these and other papers, provided solid positive pre-clinical data to warrant the initiation of Phase 1 clinical trials in 2014, of which a Phase 1 safety trial with SLC-0111 (as a monotherapeutic agent), has been completed^215^ and a Phase 1 b trial is currently underway to evaluate SLC-0111 in combination with gemcitabine in metastatic pancreatic cancer patients whose tumours are CA IX positive (ClinicalTrials.gov Identifier: NCT03450018).

Other groups also used SLC-0111 in various biomedical studies in which selective inhibition of some CA isoforms was needed. These include the effects on prostate cancer cells of SLC-0111 alone or in combination with daunorubicin^242^, radiobiological effects of CA IX inhibition in human breast cancer cells^243^, microvascular endothelial cell pH regulation^244^, glycolysis and migration suppression in pulmonary microvascular endothelial cells^245^^,^^246^, involvement of CA isoforms in mitochondrial biogenesis and in the control of lactate production in human Sertoli cells^246^. Very recently, SLC-0111 was combined with 3-O-acetylbetulin for the treatment of CA IX-positive tumours by Vordermark’s group, again with excellent synergistic effects^247^. Dedhar’s group recently reported the interconnection between CA IX inhibition and the amino acid and acid/base transporters in hypoxic tumours^248^ as well as the possibility to overcome the adaptive resistance to KRAS and MEK inhibitors by using such anticancer agents in combination with SLC-0111^249^. All these studies confirmed the usefulness of this clinical candidate, SLC-0111, in tumours and other biomedical conditions. Although Jonsson and Liljas presumably read our reply^180^, in their reply to our reply they continue that they, “would have liked to see a properly reported inhibition study of this compound to make it obvious that it indeed is a good enough inhibitor to enter Phase I/Phase II studies”^181^.

Thus, the CA field seems to be now in a typical ancient Greek tragedy situation: we have the *Deus ex machina* (the couple Jonsson and Liljas) who decide who is doing good and who is doing bad experiments (without however having any documented expertise in enzyme inhibition measurements) and then we have the *Computantis* (accountant, Matulis) who needs to check the measurements from other laboratories, and asking (in the rare occasions when he acted as reviewer to our papers) for a huge amount of supporting information data that are without any usefulness to anybody. Is this dogmatism or science?
